# Supplementary material for: Construction of an intelligent screening model for allergic rhinitis based on routine blood tests
Source: PLoS One. 2025 Dec 23;20(12):e0337561. doi: 10.1371/journal.pone.0337561 (PMC12725639; doi:10.1371/journal.pone.0337561)
Supplement: S1 Table — All features of the external validation set (minimal dataset) were statistically analyzed using SPSS. Variables with a normal distribution (including near-normal distributions) were presented as mean ± standard deviation, while non-normally distributed variables were presented as quartiles. The statistical results are shown in Table 1. Using the ensemble voting method, this study ultimately identified 16 input features. Consequently, the statistical analysis of the external validation set included only these 16 features and was conducted using SPSS software. (PDF) [file pone.0337561.s001.pdf]

Table 1. Statistical results of routine blood test data of the external validation set based on SPSS.

Characteristic	Full name	Negative and positive statistical results	Positive result	Negative result	P-value
sex		0(73.37%)	0(75.32%)	0(72.07%)	0.623
age		44.01±13.51	43.84±13.42	44.12±13.63	0.89
RBC	Red blood cell count	4.76±0.43	4.77±0.42	4.76±0.43	0.869
MCV	Mean corpuscular volume	92.15±5.97	91.77±5.99	92.4±5.97	0.485
PDW	Platelet distribution width	13.55±1.94	13.16±1.59	13.8±2.1	0.028
NEUT%	Neutrophil ratio	56.15±7.63	55.75±7.46	56.42±7.76	0.558
EO%	Eosinophil ratio	1.55(0.9,2.7)	2.4(1.35,4.)	1.2(0.7,2.)	0.532
HGB	Hemoglobin	141.93±16.84	141.49±17.36	142.23±16.57	0.774
HCT	Hematocrit	0.43±0.08	0.44±0.05	0.42±0.09	0.265
MCHC	Mean corpuscular hemoglobin concentration	323.09±11.08	322.96±12.27	323.17±10.28	0.899
RDW-CV	Red blood cell distribution width	13.34±1.32	13.36±1.45	13.32±1.24	0.816
PLT	Platelet count	258.91±62.51	251.22±60.33	263.97±63.66	0.176
MONO%	Monocyte ratio	6.41±1.49	6.39±1.58	6.42±1.44	0.887
EO#	Absolute eosinophil count	0.09(0.05,0.16)	0.13(0.07,0.25)	0.08(0.05,0.12)	0.736
RDW-SD	Red blood cell distribution width - SD	44.38±3.04	44.28±3.42	44.45±2.77	0.721

P-LCR	Large platelet ratio	30.95±8.57	30.±7.04	31.56±9.42	0.228
