# Supplementary material for: Construction of an intelligent screening model for allergic rhinitis based on routine blood tests
Source: PLoS One. 2025 Dec 23;20(12):e0337561. doi: 10.1371/journal.pone.0337561 (PMC12725639; doi:10.1371/journal.pone.0337561)
Supplement: Attachment — Submitted filename: Response to Reviewers.docx [file pone.0337561.s004.docx]

5. Review Comments to the Author

Please use the space provided to explain your answers to the questions above. You may also include additional comments for the author, including concerns about dual publication, research ethics, or publication ethics. (Please upload your review as an attachment if it exceeds 20,000 characters)

Reviewer #1: Reviewer Comments:

I. Research Value and Innovation

Clear research significance with clinical application potential. The rising prevalence of Allergic Rhinitis (AR) and the limitations of current diagnostic methods (subjectivity, high cost, invasiveness) are well-established. This study builds an intelligent screening model using routine blood test data, enabling early screening without additional tests. This holds significant practical value for primary healthcare, aligning with trends in precision medicine and AI-assisted diagnosis.

Moderate methodological innovation. The ensemble voting approach integrating multiple feature selection techniques (filter, embedded, wrapper methods) with machine learning algorithms enhances feature selection reliability and model performance compared to single methods. The soft voting ensemble strategy, weighted by AUC to optimize classifier combination, is logically sound. Results demonstrate superior model performance over single algorithms (AUC=0.862, external validation accuracy 73.91%), providing methodological reference value.

II. Method Design and Implementation

Data Source and Preprocessing

Strengths: Data originates from a single center but has a substantial sample size (n=1295). Diagnostic criteria (combining symptoms and allergen testing) are clear. Grouping methods are reasonable.

**（1）**Inclusion only of patients from Hohhot introduces geographical limitations potentially affecting model generalizability. This should be explicitly acknowledged in the Discussion.

**Response:**

We thank the reviewer for the valuable comments. As requested, we have supplemented the relevant content in the Discussion section：A notable limitation of this study is that both the model development and external validation cohorts were exclusively recruited from a single center in Hohhot. This geographical specificity may introduce bias related to the local population's genetic predispositions, predominant allergens (e.g., specific pollen types prevalent in the Inner Mongolia grassland region [6]), and regional environmental factors. Consequently, the generalizability of our screening model to populations in other geographical areas with different demographic characteristics and allergen exposures may be limited. While the internal and single-center external validation demonstrated promising performance, future validation on multi-center, geographically diverse cohorts is essential to confirm the robustness and widespread applicability of our model across various populations.In addition, this paper uses only routine blood test data when establishing the model and fails to integrate information such as contact history and symptoms of potential risk factors for AR, which reduces the accuracy and interpretability of the model. To better adapt to real clinical scenarios and improve the accuracy of decision-making, future work will aim to expand the data source and increase the information dimension of the model input.

**（2）**Details of data preprocessing (e.g., missing value handling, outlier correction, feature standardization) are omitted, potentially impacting model stability. Supplementation is recommended..

**Response:**

Thank you for your valuable feedback! We have supplemented and improved the data preprocessing process (including missing value handling, outlier handling, and feature standardization). The specific additions are as follows: the method for handling missing and outlier features has been added in line 127; the specific method and related formula for data standardization have been added in line 232.

Modify the content in line 127：“Eliminate sample data with missing values or outliers. Finally, routine examination data and AR diagnosis results were collected for 1295 subjects, including 676 AR patients and 619 non-AR patients.”

Modify the content in line 232：“ Finally, all feature data were normalized (Z-score) to eliminate the impact of dimensional differences between different detection metrics on the model. The Z-score standardization calculation formula is as follows (1):

$\text{Z=}\frac{\text{x}\text{−μ}}{\text{σ}}$	(1)”

**（3）**Specific parameter settings for the three feature selection methods (Mutual Information, LASSO, RF-RFE), such as the LASSO penalty coefficient or RF-RFE iteration count, are not detailed, hindering method reproducibility.

**Response:**

Thank you for your valuable feedback! We indeed neglected to explain the specific parameter settings for the three feature selection methods. We have now supplemented this section in the paper, located in the "Feature Selection Results" section on line 291. Specifically, the mutual information method performs feature selection by directly calculating the correlation between the data and the label, requiring no additional parameter settings. The LASSO method is based on logistic regression, and its parameters are the same as those of the logistic regression method. The RFE method is based on the random forest method, and its corresponding parameters are also the parameters of the random forest method.

Modify the content in line 291：“After multiple adjustments and optimizations, the final feature selection method and parameter settings are as follows: The filtering method (mutual information) directly calculates the correlation between feature data and labels without additional parameters, and selects the top 15 features as the screening results; the embedding method (LASSO) is built based on the logistic regression model, with the parameter configuration of penalty='l1', C=0.1, olver='liblinear', and class_weight='balanced'. The screening rule is to select features with the absolute value of the feature coefficient greater than 0; the RFE method uses the random forest model as the basic model. After parameter optimization, the random forest model parameters are set to n_estimators=10 and the class weight is 'balanced'; RFE's n_features_to_select=15 (retaining 15 important features) and step=1 (eliminating 1 feature at each iteration).”

（4）The rationale for selecting the top three base classifiers ("based on AUC value") lacks clarity regarding the threshold criterion. Supplementation with the AUC ranking results for all algorithms and statistical tests (e.g., Wilcoxon test) is recommended.

**Response:**

Thank you very much for your valuable suggestions! After careful verification, we did have the problem of insufficient explanation of the basis for selecting the three basic classifiers, and lacked clear explanation in the presentation of the results of the three feature selection methods. Based on this, we have made targeted supplements and improvements in the paper: the selection logic and theoretical support of the three basic classifiers are explained in detail on line 162; the model experimental results of the three feature selection methods are supplemented on line 331 to verify their effectiveness with objective data; the specific analysis of the feature contribution value is presented on line 311 to quantify the influence of each feature on the model performance; at the same time, the statistical test results of the input features are supplemented on line 158, and the test methods used are explained in detail on line 144 to ensure the transparency and reproducibility of the research methods. The above modifications are intended to improve the rigor and completeness of the paper. Thank you again for your professional guidance!

Modify the content in line 162：“In terms of feature selection, filtering, embedding and wrapping methods each have their own advantages: filtering uses statistical indicators (such as chi-square test and mutual information) to perform preliminary feature screening independently of the model, avoiding model assumption bias and being suitable for high-dimensional data preprocessing [14]; embedding embeds feature selection into the model training process (such as LASSO or feature importance of tree models), automatically eliminates redundant features through regularization or integration mechanisms, and is particularly suitable for processing nonlinear relationship features [15,16]; wrapping is guided by model performance (such as recursive feature elimination, RFE), and captures feature interaction effects through iterative search, and performs outstandingly in scenarios such as medical diagnosis that require fine optimization of feature combinations [17]. Based on the above advantages, this study selected these three methods for feature screening experiments.”

Modify the content in line 331：“Table 3. Comparison of Classification Performance of SVM Models on Different Feature Sets

| Evaluation  Metrics  Feature Set | AUC | Accuracy | Recall | Specificity |
| --- | --- | --- | --- | --- |
| Raw Features | 0.822 | 73.75% | 75.56% | 71.77% |
| Mutual Information | 0.835 | 75.68% | 74.81% | 76.61% |
| RF-REF | 0.825 | 74.13% | 75.56% | 72.58% |
| LASSO | 0.825 | 74.90% | 74.07% | 75.81% |
| Core Features | 0.845 | 77.61% | 75.56% | 79.84% |

”

Modify the content in line 311：“Table 2. Feature screening results based on filtering, wrapping, and embedding methods

| Feature selection method | Filtering (mutual information) | | Wrapping (REF) | | Embedding (LASSO) | |
| --- | --- | --- | --- | --- | --- | --- |
|  | Feature Name | Contribution value | Feature Name | Contribution value | Feature Name | Contribution value |
| 1 | EO# | 0.079 | RDW-SD | 0.102 | RDW-CV | 0.315 |
| 2 | RDW-SD | 0.063 | EO% | 0.101 | MPV | 0.132 |
| 3 | EO% | 0.052 | EO# | 0.097 | RDW-SD | -0.129 |
| 4 | age | 0.034 | age | 0.091 | sex | 0.115 |
| 5 | MCH | 0.029 | HCT | 0.081 | PDW | -0.080 |
| 6 | RBC | 0.027 | PDW | 0.060 | EO% | 0.069 |
| 7 | RDW-CV | 0.024 | MCHC | 0.056 | MCV | 0.052 |
| 8 | sex | 0.022 | MCV | 0.054 | MONO% | -0.008 |
| 9 | MCHC | 0.022 | PLT | 0.053 | P-LCR | -0.008 |
| 10 | WBC | 0.015 | NEUT% | 0.053 | age | -0.007 |
| 11 | HCT | 0.014 | LYMPH# | 0.052 | NEUT% | 0.004 |
| 12 | PDW | 0.013 | HGB | 0.051 | HGB | -0.003 |
| 13 | NEUT% | 0.012 | RDW-CV | 0.051 | PLT | -0.001 |
| 14 | MONO% | 0.011 | RBC | 0.050 | MCHC | 0.000 |
| 15 | P-LCR | 0.010 | NEUT# | 0.050 |  |  |

”

Modify the content in line 144：“Before model construction, to optimize the effect of feature screening, this study used SPSS software for statistical analysis based on 28-dimensional features (26 routine blood test indicators and age and gender). The statistical methods selected were as follows: (1) Qualitative variables were tested using the chi-square test and expressed as categories (percentages); (2) Quantitative variables were first tested for normality. If the variables were normal (Shapiro-Wilk test p>0.05) or approximately normal distribution (standard deviation < mean/3), the independent sample t-test (mean ± standard deviation) was used, and significance was determined by the Levene test (Student's t-test was used when p>0.05, otherwise the Welch's t-test was used); (3) Non-normal or approximately normal distribution variables were tested using the Mann-Whitney U test and expressed as median (upper quartile, lower quartile). Specific feature abbreviations, definitions, and statistical results are shown in Table 1.”

Modify the content in line 158：“Table 1. Statistical results of blood routine test data based on SPSS.

| Characteristic | Full name | Negative and positive statistical results | Positive result | Negative result | P-value |
| --- | --- | --- | --- | --- | --- |
| sex |  | 0(61.69%) | 0(75.61%) | 0(68.34%) | <0.001 |
| age |  | 43.38±11.74 | 40.65±10.17 | 46.36±12.59 | <0.001 |
| RBC | Red blood cell count | 4.8±0.46 | 4.8±0.47 | 4.81±0.45 | 0.687 |
| MCV | Mean corpuscular volume | 92.02±5.46 | 91.84±4.82 | 92.21±6.09 | 0.224 |
| PDW | Platelet distribution width | 13.51±1.8 | 13.3±1.71 | 13.73±1.87 | <0.001 |
| WBC | White blood cell count | 6.34±1.6 | 6.37±1.57 | 6.32±1.64 | 0.528 |
| NEUT% | Neutrophil ratio | 56.69±7.79 | 56.77±7.73 | 56.61±7.87 | 0.706 |
| LYMPH% | Lymphocyte ratio | 33.99±7.42 | 33.35±7.22 | 34.69±7.58 | 0.001 |
| EO% | Eosinophil ratio | 1.7(1.,3.) | 2.4(1.4,3.9) | 1.3(0.8,2.) | <0.001 |
| BASO% | Basophil ratio | 0.5(0.3,0.6) | 0.5(0.3,0.6) | 0.5(0.3,0.6) | 0.459 |
| NEUT# | Neutrophil absolute value | 3.49(2.8,4.26) | 3.66±1.21 | 3.49(2.76,4.26) | 0.385 |
| BASO# | Absolute basophil count | 0.03(0.02,0.04) | 0.03(0.02,0.04) | 0.03(0.02,0.04) | 0.408 |
| HGB | Hemoglobin | 143.23±17.02 | 143.03±16.46 | 143.44±17.62 | 0.671 |
| HCT | Hematocrit | 0.42±0.11 | 0.44±0.04 | 0.43(0.4,0.47) | <0.001 |
| MCH | Mean corpuscular hemoglobin content | 29.81±2.15 | 29.81±1.9 | 29.82±2.39 | 0.912 |
| MCHC | Mean corpuscular hemoglobin concentration | 323.88±11.42 | 324.49±10.49 | 323.22±12.33 | 0.047 |
| RDW-CV | Red blood cell distribution width | 13.37±1.48 | 13.19±1.41 | 13.57±1.53 | <0.001 |
| PLT | Platelet count | 256.9±60.81 | 254.34±55.5 | 259.69±66.06 | 0.117 |
| MPV | Mean platelet volume | 11.13±1.21 | 11.11±1.23 | 11.15±1.2 | 0.605 |
| PCT | Platelet count | 0.28±0.06 | 0.28±0.06 | 0.29±0.07 | 0.099 |
| MONO# | Absolute value of monocytes | 0.4±0.13 | 0.4±0.13 | 0.41±0.14 | 0.634 |
| MONO% | Monocyte ratio | 6.42±1.5 | 6.38±1.47 | 6.48±1.53 | 0.219 |
| EO# | Absolute eosinophil count | 0.11(0.06,0.18) | 0.15(0.08,0.23) | 0.08(0.05,0.13) | <0.001 |
| IG# | Immature granulocyte count | 0.01(0.01,0.01) | 0.01(0.01,0.01) | 0.01(0.01,0.01) | 0.484 |
| RDW-SD | Red blood cell distribution width - SD | 44.16±3.57 | 43.22±3.48 | 45.18±3.39 | <0.001 |
| IG% | The percentage of immature granulocytes | 0.1(0.1,0.2) | 0.1(0.1,0.2) | 0.1(0.1,0.2) | 0.843 |
| P-LCR | Large platelet ratio | 30.78±7.85 | 30.37±7.56 | 31.23±8.14 | 0.049 |
| LYMPH# | Absolute lymphocyte count | 2.12±0.57 | 2.08±0.54 | 2.15±0.6 | 0.034 |

”

**（5）**The source, sample size, and feature distribution of the external validation set are not specified. Clarification is needed on whether it is an independent cohort or cross-center data.

**Response:**

Thank you for the valuable comments provided by the reviewers. We have supplemented the detailed information of the external validation set as requested and made the corresponding revisions in the main text.

Modify the content of line 131：“This study used a single-center prospectively recruited external validation cohort. The data came from outpatients in the Allergy Department of Hohhot First Hospital from March 21 to September 3, 2023. After screening and meeting the inclusion criteria, a total of 184 patients aged 19-70 years (49 males and 135 females) were included; according to clinical gold standards (such as allergen sIgE testing and typical symptoms and signs), they were divided into 73 allergic rhinitis (AR) patients and 111 non-AR control groups.”

**（6）**Comparison with single algorithms is limited to AUC and accuracy. The absence of a calibration curve or Decision Curve Analysis (DCA) makes it difficult to comprehensively evaluate clinical net benefit.

**Response:**

Thank you for your suggestions. Regarding model calibration analysis, inherent limitations of the dataset currently present significant challenges. Although the models generalize well in training and testing, the ensemble models (SVM, LR, and RF) struggle to correct for probability bias due to inappropriate calibration methods. Furthermore, the boundary between AR and healthy individuals is blurred (e.g., it's difficult to distinguish between mild cases and those with allergies). Some "gray zone" samples lead to fluctuations in predictions within the intermediate probability range (0.4–0.6), causing the calibration curve to deviate from the ideal line. Furthermore, the limited amount of available calibration data results in low stability and a lack of true reflection of test set performance. Therefore, the current calibration plots are ineffective. We plan to reanalyze the data distribution through data augmentation, SMOTE, or an external validation set to improve the data distribution. Furthermore, this study pre-determined a classification threshold of 0.5 to correspond to clinical treatment decisions (positive, recommend treatment; negative, no recommendation or further testing). Because AR treatment is low-risk, reversible, and has mild adverse reactions, the clinical significance of assessing the benefit-risk trade-off at different thresholds is limited, so a decision curve analysis (DCA) was not performed.

（7） Feature importance ranking lacks discussion in the context of clinical significance (e.g., were eosinophil absolute count/percentage assigned the highest weights?). Supplementation with feature contribution analysis (e.g., SHAP values or Permutation Importance) is recommended.

**Response:**

Sincerely thank you for your valuable suggestions. Given that this experiment utilizes an ensemble model constructed from three optimal models, to more comprehensively and deeply illustrate the model's decision-making mechanism and feature importance, this study supplements the SHAP plots and detailed analysis corresponding to these three optimal classification models. The clinical significance of feature importance is also discussed. The supplementary SHAP plots are detailed in line 379 of the paper.

Modify the content in 380:“ The Support Vector Machine (SVM) model assigned the highest importance to RDW-SD, PDW, sex, P-LCR, and EO% (Figures 5A, 5B). RDW-SD (Red Blood Cell Distribution Width - Standard Deviation): This was identified as the most important feature in the SVM model. RDW-SD quantifies the heterogeneity in red blood cell size (anisocytosis). While not a traditional marker for AR, elevated RDW is increasingly recognized as a surrogate marker of systemic inflammation. The chronic inflammatory state in AR could potentially influence erythropoiesis, leading to greater variation in red cell size. In our analysis, higher RDW-SD values (red) correlated with increased SHAP values, suggesting a positive contribution to an AR prediction. PDW (Platelet Distribution Width) & P-LCR (Platelet Large Cell Ratio): These parameters measure the variability in platelet size. Larger, more reactive platelets are often associated with inflammatory and immune-mediated conditions. Platelets are known to participate in allergic inflammation by releasing mediators and interacting with other immune cells. The importance of PDW and P-LCR in our model suggests a potential link between platelet activation and AR. The SHAP dependence plots show that elevated levels of these markers (red) contribute to a higher probability of AR. The inclusion of sex as a key feature is supported by well-documented epidemiological evidence. The prevalence and severity of allergic diseases, including AR, often exhibit significant differences between males and females, likely due to a complex interplay of hormonal, genetic, and environmental factors. Our model has captured this effect, with the specific SHAP value for each patient indicating the direction and magnitude of sex's contribution to their individual prediction. EO% (Eosinophil Percentage): This feature was among the model's top contributors, which is highly consistent with established clinical pathophysiology. Eosinophils are the principal effector cells in allergic inflammation. An elevated EO% is a canonical biomarker for type 2 immune responses, directly reflecting allergic sensitization and disease activity in AR [43]. In the SHAP plot, higher values of EO% (shown in red) are strongly associated with positive SHAP values, meaning they push the model's prediction towards a positive AR diagnosis. This robust alignment with gold-standard clinical knowledge strongly validates our model's biological plausibility.

The Random Forest (RF) model identified RDW-SD and EO# among its most influential features, followed by EO% (Figures 5C, 5D). The reaffirmation of eosinophil-related parameters (EO#, EO%) across all models underscores their critical and non-redundant role in AR screening. The concomitant importance of RDW-SD across all three models suggests a previously underappreciated link between erythrocyte indices and allergic inflammation that warrants further investigation; In the Logistic Regression (LR) model, the top contributing features were RDW-SD, RDW-CV, and PDW(Figures 5E, 5F). Elevated red cell distribution width (RDW) values, indicating heterogeneity in red blood cell size (anisocytosis), have been increasingly associated with systemic inflammatory states. In the context of AR, a chronic inflammatory condition, elevated RDW may serve as a surrogate marker for underlying inflammatory processes. Similarly, Mean Platelet Volume (MPV) is a marker of platelet activation, which can be modulated by allergic and inflammatory responses.

In summary, the feature contribution analysis confirms that our ensemble model successfully leverages features with strong biological and clinical foundations. The prominence of eosinophil counts (EO#, EO%) directly corroborates the pathophysiological mechanism of AR, thereby enhancing the clinical interpretability and credibility of our intelligent screening tool. The recurring importance of RDW-related features points to a potential novel hematological dimension in AR that merits future research.

Figure 5. SHAP interpretability analysis of the three best models: support vector machine (A, B), random forest (C, D), and logistic regression (E, F).

”

（8） Table 2 (Parameter Configuration) lacks complete presentation of some algorithm parameters (e.g., SVM kernel function, KNN neighbor count), hindering model replication.

**Response:**

We sincerely thank you for your careful reminder and valuable suggestions. We have comprehensively improved the characteristic parameter settings of the model in Table 4. For hyperparameters not listed, the model default values are used to ensure the standardization and reproducibility of the experiment.

Modify the content in 340：“Table 4.Model parameter setting.

| algorithm | parameter | value |
| --- | --- | --- |
| KNN | n_neighbors | 10 |
|  | weights | uniform |
|  | algorithm | auto |
|  | p | 2 |
| LR | penalty | l2 |
|  | C | 1 |
|  | max_iter | 100 |
|  | solver | sag |
|  | random_state | 168 |
| DT | criterion | gini |
|  | splitter | random |
|  | max_features | auto |
|  | max_depth | 15 |
|  | random_state | 84 |
| RF | criterion | gini |
|  | n_estimators | 90 |
|  | max_features | auto |
|  | random_state | 84 |
| SVM | kernel | rbf |
|  | C | 1.4 |
|  | gamma | 0.1 |
|  | coef0 | 0 |
|  | random_state | 42 |

”

**（9）**The current figure legend for the confusion matrix (likely Fig 5) lists only "False positive," "False negative," and "Correct prediction" categories without specific numerical values or percentages. Adding this data would enhance clarity. Terminology must be consistent with definitions in the main text. Briefly define these terms in the legend or text. Color legends (■) must correspond precisely to chart colors. If figures are for potential B&W printing, use distinct patterns (e.g., hatching, dots) instead.

**Response:**

We sincerely thank you for your valuable suggestions. After careful review, we realize that simply labeling the figures with numbers can hinder reader comprehension. To improve the clarity and readability of our research, we have supplemented the paper with all experimental results from the external test set and the corresponding confusion matrix.

The detailed modification content is in line 412：“The performance evaluation results of the AR early screening model based on the ensemble voting method on the external validation set are shown in Table 7. Compared with the actual clinical diagnosis results, the model showed good screening effect, with an accuracy of 0.739, an AUC of 0.722, and a high specificity (0.829). It was reliable in excluding non-cases, with a recall rate of 0.603 and a medium sensitivity, which can meet the basic clinical needs of early screening. The confusion matrix of the external validation set is shown in Figure 6, which contains 184 samples. The model predicted 92 true negatives (TN), 19 false positives (FP), 29 false negatives (FN), and 44 true positives (TP). The total number of correctly predicted samples was 136, corresponding to an accuracy of 0.739.

Table 7. Evaluation metrics of the external validation set.

| Evaluation Metrics | Data Value | Evaluation Metrics | Data Value |
| --- | --- | --- | --- |
| AUC | 0.722 | TN | 92 |
| Accuracy | 0.739 | FP | 19 |
| Recall | 0.603 | FN | 29 |
| Specificity | 0.829 | TP | 44 |

Figure 6. External validation set performance evaluation results: (A) confusion matrix and (B) classification performance index analysis.

”

The term supplement is on line 246：“In the evaluation of medical diagnostic models, each indicator has clear clinical significance: AUC is a comprehensive indicator of the overall discriminative efficiency of the model, and its value range of 0.5-1.0 reflects the model's ability to distinguish between disease and non-disease states. The closer the value is to 1, the better the discriminative efficiency; Accuracy represents the overall proportion of correct predictions of all samples by the model; Recall specifically refers to the proportion of correctly identified samples in actual positive samples, reflecting the disease detection ability; Specificity indicates the proportion of correctly excluded samples in actual negative samples, reflecting the accuracy of disease exclusion. True positive (TP, actually positive and correctly predicted), true negative (TN, actually negative and correctly predicted), false positive (FP, actually negative but incorrectly predicted as positive) and false negative (FN, actually positive but incorrectly predicted as negative), these basic indicators constitute the confusion matrix for model performance evaluation. ”

**（10）**Regarding data presentation: The meaning of numbers like "136, 19, 29" below charts is unclear (possibly sample counts per category or other metrics). Explicit labeling is required to prevent misinterpretation. If sample counts, supplement with relevant statistical metrics (e.g., accuracy, recall) to bolster result credibility.

**Response:**

We have provided detailed experimental results for the external validation set. The number 139 represents the number of samples correctly predicted by the model. We have also provided clear explanations of the data within the charts. Detailed test set evaluation results are provided in Table 7 on line 419 of the paper.

The modification is in line 419: “The confusion matrix of the external validation set is shown in Figure 6, which contains 184 samples. The model predicted 92 true negatives (TN), 19 false positives (FP), 29 false negatives (FN), and 44 true positives (TP). The total number of correctly predicted samples was 136, corresponding to an accuracy of 0.739.”

**（11）**Formatting & Norms: Figure filenames (e.g., Fig5.tif) should follow journal style (e.g., "Figure5.tif" or "Fig5.png"). Ensure figure resolution meets journal requirements (typically ≥300 dpi).

**Response:**

Thank you very much for your valuable suggestions. We have re-saved all images according to professional standards. All images are in PNG format and the resolution has been uniformly increased to no less than 300dpi to ensure the clarity and quality of the images in print and electronic display.

（12）Insufficient comparison with similar studies (e.g., Christo et al.'s 97.7% accuracy model). Analyze methodological differences (e.g., data type, algorithm choice) to clarify this model's specific applicability.

**Response:**

We thank the reviewer for the valuable comments. As requested, we have supplemented the relevant content in the Discussion section.

The supplement is on line 507: “Our model achieved an accuracy of 73.91% upon external validation, which is lower than the 97.7% accuracy reported by Christo et al. [45] using a genetic algorithm and extreme learning machine. This discrepancy can be primarily attributed to fundamental methodological differences. The study by Christo et al. likely utilized a feature set that included specific allergen test results or detailed symptom profiles, which are highly predictive but less universally available. In contrast, our model relies solely on routine blood test data, a more accessible and cost-effective but also more general and nonspecific data source. This strategic choice inherently trades off some predictive power for significantly greater applicability in primary care and resource-limited settings where advanced allergen testing is not feasible. Therefore, our model is not intended to replace specialized diagnostic tests but rather to serve as a widespread, first-line screening tool. Similarly, compared to the model by Kavya et al. [46] (accuracy: 86.39%), which incorporated clinical symptom data, our blood-based model offers the advantage of objectivity, minimizing the subjectivity associated with patient-self-reported symptoms.”

**（13）**The impact of dynamic changes in blood routine indicators (e.g., seasonal fluctuations in seasonal AR patients) on the model is not explored and could be a valuable extension.

**Response:**

We thank the reviewer for the valuable comments. As requested, we have supplemented the relevant content in the Discussion section.

The additional content is on line 540: “This study utilized routine blood test data from a single time point, which does not account for the dynamic physiological changes inherent to allergic conditions. For instance, eosinophil counts (%)—a key feature in our model—are known to fluctuate with allergen exposure intensity, such as seasonal pollen variations [6, 43]. A model trained on static measurements may therefore be less reliable for patients whose blood parameters are in a transient state. This limitation highlights a valuable direction for future research. Subsequent studies could implement longitudinal tracking of blood parameters in AR patients across different seasons. Developing a model that incorporates temporal patterns of change or that recommends diagnosis based on tests performed during symptomatic periods could significantly enhance predictive accuracy and clinical utility for seasonal AR sufferers.”

**（14）**Ethical Compliance. Mention of Institutional Review Board (IRB) approval is noted, but details regarding patient informed consent and data anonymization procedures are missing. Supplementation of the ethics statement details is required.

**Response:**

We sincerely thank the reviewer for this important reminder. We have now added a detailed ethics statement in the "Data Sources" section, line 103. Informed consent was obtained from all participants after a detailed explanation of the study.All personal identifiable information was removed, and a full anonymization procedure was implemented to ensure data privacy.We apologize for the oversight and appreciate the opportunity to improve the manuscript's compliance with ethical reporting standards.

Edition in line 103:“The study was approved by the Ethics Committee of Hohhot First Hospital(Approval No: [IRB2022018]). All procedures performed in studies involving human participants were in accordance with the ethical standards of the institutional and/or national research committee and with the 1964 Helsinki declaration and its later amendments or comparable ethical standards. Informed consent was obtained from all individual participants included in the study. Prior to data collection, the purpose of the study, potential risks and benefits, and the confidentiality of their information were explained in detail to all participants. To ensure privacy and confidentiality, all personally identifiable information (PII) of participants was removed during data preprocessing. This included, but was not limited to, names, identification numbers, and contact information. Each patient was assigned a unique, non-identifiable code for data analysis. ”

**（16）**Some figure/table labels are unclear (e.g., missing y-axis unit in Fig 3, inconsistent table numbering). Standardize figure/table formatting.

**Response:**

Thank you very much for your valuable feedback! We have carefully reviewed all figures in accordance with your suggestions. We have not only ensured that each table has clear and accurate X-axis and Y-axis labels to ensure the standardization and readability of the data presentation, but also carefully checked the numbering of tables and figures to avoid confusion or omissions, thereby maintaining the rigor and professionalism of the research.

**（17）**Redundant descriptions exist between the English abstract and main text (e.g., repeated steps of the ensemble voting in the Methods). Streamlining is advised.

**Response:**

Thank you very much for your valuable suggestions! We have rewritten the methods section. Since both the feature selection and model building stages use an ensemble voting method, there are differences between the two: the feature selection stage uses a hard voting strategy and is based on three feature selection methods; the model building stage uses five machine learning methods and is based on an ensemble voting soft voting mechanism. To help readers deeply understand the research methods and ensure the reproducibility of the code, we need to explain in detail the reasons for choosing the feature selection method and the model building method. Based on this, we have completely rewritten the experimental methods section. While ensuring the completeness of the content, we have simplified the sentences as much as possible to improve the conciseness and professionalism of the text.

The modification is in line 162 of the paper: “In terms of feature selection, filtering, embedding and wrapping methods each have their own advantages: filtering uses statistical indicators (such as chi-square test and mutual information) to perform preliminary feature screening independently of the model, avoiding model assumption bias and being suitable for high-dimensional data preprocessing [14]; embedding embeds feature selection into the model training process (such as LASSO or feature importance of tree models), automatically eliminates redundant features through regularization or integration mechanisms, and is particularly suitable for processing nonlinear relationship features [15,16]; wrapping is guided by model performance (such as recursive feature elimination, RFE), and captures feature interaction effects through iterative search, and performs outstandingly in scenarios such as medical diagnosis that require fine optimization of feature combinations [17]. Based on the above advantages, this study selected these three methods for feature screening experiments.

This study proposes a robust feature selection method based on an integrated voting strategy, which constructs an optimal feature subset by integrating the advantages of filtering, embedding, and wrapping. The specific implementation is divided into two stages: the first stage adopts multi-strategy parallel screening, in which the filtering method selects the top 15 important features based on mutual information [18], the embedding method selects non-zero coefficient features through LASSO regression [19,20], and the wrapping rule uses recursive feature elimination (RFE) combined with a random forest model to retain the top 15 important features [21-23]. The second stage integrates the three sets of candidate features through a hard voting mechanism, and determines the features with a screening frequency ≥ 2 as the final feature subset. This method innovatively combines the model independence of the filtering method, the regularization constraint of the embedding method, and the performance-oriented characteristics of the wrapping method. Through the majority voting mechanism, it effectively improves the stability and generalization ability of feature selection, and is particularly suitable for ensemble learning scenarios where the prediction performance of the base models is similar but there are differences [24].

This study uniformly uses support vector machines (SVMs) as classification models. The ability of its kernel function to process high-dimensional feature spaces effectively avoids the dimensionality curse problem and is particularly suitable for verifying feature subsets after filtering and embedding methods. Based on the principle of structural risk minimization, SVM gives the model natural robustness to feature redundancy and noise, and can objectively evaluate the changes in the generalization performance of the wrapping method during iterative feature elimination. In order to comprehensively evaluate the effect of feature selection, indicators such as the area under the ROC curve (AUC), accuracy (ACC), specificity (SPE), and sensitivity (SEN) [25] are used to systematically compare the performance of filtering, embedding, wrapping, and ensemble voting methods in feature selection.”

Reviewer #2: I agree that there are few objective tests for allergic rhinitis and that this poses several challenges.

I have a few comments

**（1）**I have reservations about whether the blood tests presented in the study can be fully conducted at primary care facilities rather than tertiary hospitals. Screening tests for allergic rhinitis should ideally be low-cost and accessible at the primary care level.

**Response:**

The experimental data used in this study were primarily based on routine blood tests. This test, a fundamental and essential clinical procedure in China, has been fully implemented at all levels of primary healthcare institutions, including urban community health service centers, township health centers, and village clinics. The high penetration rate of testing equipment means that screening can be conducted without additional hardware costs. Routine blood tests are simple to perform. Whether collecting blood from a fingertip or venous source, these tests are core skills that primary healthcare professionals are proficient in, requiring no specialized training. Furthermore, the tests are inexpensive, accessible, and well-accepted. In the primary healthcare setting, the greatest advantage of developing a screening model based on routine blood tests is that they perfectly align with the primary screening phase of the "screening-diagnosis-treatment" system. Leveraging existing equipment, personnel, and medical insurance policies, a comprehensive early screening network for allergic rhinitis can be established at near-zero marginal cost. This strategy not only helps increase the early detection rate of allergic rhinitis (AR), but also promotes the implementation of tiered diagnosis and treatment, effectively alleviating the burden on tertiary hospitals. It represents a practical, efficient solution tailored to China's national conditions. Therefore, routine blood tests, due to their widespread availability, affordability, and operability, are well-suited as a basic data source for disease screening in primary healthcare settings.

**（2）**The use of hard and soft voting methods combining KNN, LR, RF, DT, and SVM models appears to yield improved results compared to previous approaches. However, the accuracy rate of 73.91% for a screening test requires cautious interpretation. It is recommended that additional clarification or discussion of the limitations be provided to contextualize this result appropriately.

**Response:**

On the external validation set, the model achieved a precision of 0.739, a specificity of 0.829, and a recall of 0.603. The results on the external validation set suggest that the model exhibits both strengths and weaknesses. However, analysis of the test results during the training phase (specificity: 0.855, precision: 0.772, recall: 0.696) indicates that the model actually exhibits relatively good predictive performance. The data distribution of the external validation set differs from the training data, which to some extent limits the model's generalization ability. Insufficient sample size may also contribute to this issue. Due to the limited sample size, it is difficult to fully capture the overall population characteristics, which in turn affects the model's performance on the external validation set. We have supplemented the experimental results on the external validation set in line 384 of the paper and further elaborated on the limitations of the experiment in the Discussion section.

The modification is in line 413: “The performance evaluation results of the AR early screening model based on the ensemble voting method on the external validation set are shown in Table 7. Compared with the actual clinical diagnosis results, the model showed good screening effect, with an accuracy of 0.739, an AUC of 0.722, and a high specificity (0.829). It was reliable in excluding non-cases, with a recall rate of 0.603 and a medium sensitivity, which can meet the basic clinical needs of early screening. The confusion matrix of the external validation set is shown in Figure 6, which contains 184 samples. The model predicted 92 true negatives (TN), 19 false positives (FP), 29 false negatives (FN), and 44 true positives (TP). The total number of correctly predicted samples was 136, corresponding to an accuracy of 0.739.”

The additional content is on line 507: “Our model achieved an accuracy of 73.91% upon external validation, which is lower than the 97.7% accuracy reported by Christo et al. [45] using a genetic algorithm and extreme learning machine. This discrepancy can be primarily attributed to fundamental methodological differences. The study by Christo et al. likely utilized a feature set that included specific allergen test results or detailed symptom profiles, which are highly predictive but less universally available. In contrast, our model relies solely on routine blood test data, a more accessible and cost-effective but also more general and nonspecific data source. This strategic choice inherently trades off some predictive power for significantly greater applicability in primary care and resource-limited settings where advanced allergen testing is not feasible. Therefore, our model is not intended to replace specialized diagnostic tests but rather to serve as a widespread, first-line screening tool. Similarly, compared to the model by Kavya et al. [46] (accuracy: 86.39%), which incorporated clinical symptom data, our blood-based model offers the advantage of objectivity, minimizing the subjectivity associated with patient-self-reported symptoms.”

**（3）**A variety of inference models are employed, and the selection process for these models is considered important. Although numerous references are cited, it would be beneficial to include a clear rationale for the selection of KNN, LR, RF, DT, and SVM. While the performance evaluation and validation process using voting methods is illustrated schematically, the presented models appear to be basic validation models. Therefore, it would be helpful to provide an explanation addressing the absence of more recent models to enhance the study's relevance and rigor.

**Response:**

Thank you for your suggestion. We have added the reasons for choosing five machine learning models, namely K-nearest neighbor, logistic regression, random forest, decision tree and SVM, for the experiment in the paper. The K-nearest neighbor method does not require complex modeling and is easy to understand and implement. Doctors can intuitively judge the disease based on similar patient indicators. It has good adaptability to nonlinear data, does not rely on data distribution assumptions, and can handle complex patterns and nonlinearly separable data. Logistic regression can give the contribution coefficient of each blood routine indicator to the classification result, helping doctors understand the size and direction of the indicator's influence, which is conducive to understanding the disease mechanism and diagnostic basis. It has fast training and prediction speed for blood routine data with low dimensions, and is suitable for time-sensitive scenarios such as clinical diagnosis. Random forest integrates multiple decision trees to reduce the risk of overfitting, better handle data noise and anomalies, improve generalization ability, and automatically process multiple features and evaluate their importance. The decision tree generates an intuitive tree structure, and doctors can clearly see the decision process and feature division criteria, which is convenient for displaying the binary diagnosis process based on blood routine indicators in an easy-to-understand way, and is convenient for interactive explanation with the model. Although the dimensions of blood routine indicators are not high, there may be complex nonlinear relationships. SVM By implicitly mapping data to a high-dimensional space through kernel functions, such relationships can be better processed to improve classification accuracy. Its goal of maximizing the classification interval makes the classification results robust, better adaptable to new data, reduces overfitting, and has good generalization performance in actual disease diagnosis.

The modification is in line 212 of the paper: “These five machine learning algorithms have the following characteristics for binary classification: KNN (K-Nearest Neighbor) calculates the distance between samples and uses majority voting among neighbors for classification. It is suitable for complex data distributions and sensitive to local patterns; LR (Logistic Regression) uses the sigmoid function to achieve probability mapping and has excellent interpretability; RF (Random Forest) uses bootstrap sampling to construct multiple decision trees and ensemble voting, effectively handling feature interactions; DT (Decision Tree) recursively partitions the feature space based on information gain, generating interpretable tree-like decision rules; SVM (Support Vector Machine) constructs an optimal hyperplane by maximizing the classification margin. Its kernel function can handle nonlinear problems and is robust to small sample sizes. These algorithms provide different solutions to classification problems from different perspectives. The AUC value is used as the evaluation indicator to compare the five machine learning algorithms, and the three methods with the highest AUC values are selected to build an integrated model.”

**（4）**While utilizing the abundant data available in China is advantageous, considering that academic journals are read by a global audience, it would be more beneficial to frame the study from a perspective that offers utility to all patients, rather than focusing solely on advancements in the Chinese healthcare system.

**Response:**

This study constructed a prediction model based on routine blood test indicators. Although its application scenario is based on China's primary medical system, which has the unique advantages of comprehensive popularization of routine blood tests, low cost and simple operation, the significance of this study is not limited to serving the optimization of China's medical system. More broadly, the results of this study verified the important role and potential value of routine blood tests, a basic test data, in the screening of allergic rhinitis (AR), and provided a replicable methodological reference and empirical basis for the use of routine test items for early disease screening worldwide, especially in areas with limited medical resources. Therefore, this study not only reflects practical innovation in the specific medical context of China, but also contributes universal experience to improving the efficiency of disease screening based on basic medical data worldwide.

**（5）**It would be nice to add demographic data

**Response:**

Thank you very much for your valuable suggestions! Since our experiment incorporated key demographic variables (gender and age), we have supplemented the paper with SPSS statistical analysis of the experimental data to more clearly and intuitively present the distribution of data characteristics and enhance the credibility and interpretability of the research results.

Supplementary content is on line 144: “Before model construction, to optimize the effect of feature screening, this study used SPSS software for statistical analysis based on 28-dimensional features (26 routine blood test indicators and age and gender). The statistical methods selected were as follows: (1) Qualitative variables were tested using the chi-square test and expressed as categories (percentages); (2) Quantitative variables were first tested for normality. If the variables were normal (Shapiro-Wilk test p>0.05) or approximately normal distribution (standard deviation < mean/3), the independent sample t-test (mean ± standard deviation) was used, and significance was determined by the Levene test (Student's t-test was used when p>0.05, otherwise the Welch's t-test was used); (3) Non-normal or approximately normal distribution variables were tested using the Mann-Whitney U test and expressed as median (upper quartile, lower quartile). Specific feature abbreviations, definitions, and statistical results are shown in Table 1.

Table 1. Statistical results of blood routine test data based on SPSS.

| Characteristic | Full name | Negative and positive statistical results | Positive result | Negative result | P-value |
| --- | --- | --- | --- | --- | --- |
| sex |  | 0(61.69%) | 0(75.61%) | 0(68.34%) | <0.001 |
| age |  | 43.38±11.74 | 40.65±10.17 | 46.36±12.59 | <0.001 |
| RBC | Red blood cell count | 4.8±0.46 | 4.8±0.47 | 4.81±0.45 | 0.687 |
| MCV | Mean corpuscular volume | 92.02±5.46 | 91.84±4.82 | 92.21±6.09 | 0.224 |
| PDW | Platelet distribution width | 13.51±1.8 | 13.3±1.71 | 13.73±1.87 | <0.001 |
| WBC | White blood cell count | 6.34±1.6 | 6.37±1.57 | 6.32±1.64 | 0.528 |
| NEUT% | Neutrophil ratio | 56.69±7.79 | 56.77±7.73 | 56.61±7.87 | 0.706 |
| LYMPH% | Lymphocyte ratio | 33.99±7.42 | 33.35±7.22 | 34.69±7.58 | 0.001 |
| EO% | Eosinophil ratio | 1.7(1.,3.) | 2.4(1.4,3.9) | 1.3(0.8,2.) | <0.001 |
| BASO% | Basophil ratio | 0.5(0.3,0.6) | 0.5(0.3,0.6) | 0.5(0.3,0.6) | 0.459 |
| NEUT# | Neutrophil absolute value | 3.49(2.8,4.26) | 3.66±1.21 | 3.49(2.76,4.26) | 0.385 |
| BASO# | Absolute basophil count | 0.03(0.02,0.04) | 0.03(0.02,0.04) | 0.03(0.02,0.04) | 0.408 |
| HGB | Hemoglobin | 143.23±17.02 | 143.03±16.46 | 143.44±17.62 | 0.671 |
| HCT | Hematocrit | 0.42±0.11 | 0.44±0.04 | 0.43(0.4,0.47) | <0.001 |
| MCH | Mean corpuscular hemoglobin content | 29.81±2.15 | 29.81±1.9 | 29.82±2.39 | 0.912 |
| MCHC | Mean corpuscular hemoglobin concentration | 323.88±11.42 | 324.49±10.49 | 323.22±12.33 | 0.047 |
| RDW-CV | Red blood cell distribution width | 13.37±1.48 | 13.19±1.41 | 13.57±1.53 | <0.001 |
| PLT | Platelet count | 256.9±60.81 | 254.34±55.5 | 259.69±66.06 | 0.117 |
| MPV | Mean platelet volume | 11.13±1.21 | 11.11±1.23 | 11.15±1.2 | 0.605 |
| PCT | Platelet count | 0.28±0.06 | 0.28±0.06 | 0.29±0.07 | 0.099 |
| MONO# | Absolute value of monocytes | 0.4±0.13 | 0.4±0.13 | 0.41±0.14 | 0.634 |
| MONO% | Monocyte ratio | 6.42±1.5 | 6.38±1.47 | 6.48±1.53 | 0.219 |
| EO# | Absolute eosinophil count | 0.11(0.06,0.18) | 0.15(0.08,0.23) | 0.08(0.05,0.13) | <0.001 |
| IG# | Immature granulocyte count | 0.01(0.01,0.01) | 0.01(0.01,0.01) | 0.01(0.01,0.01) | 0.484 |
| RDW-SD | Red blood cell distribution width - SD | 44.16±3.57 | 43.22±3.48 | 45.18±3.39 | <0.001 |
| IG% | The percentage of immature granulocytes | 0.1(0.1,0.2) | 0.1(0.1,0.2) | 0.1(0.1,0.2) | 0.843 |
| P-LCR | Large platelet ratio | 30.78±7.85 | 30.37±7.56 | 31.23±8.14 | 0.049 |
| LYMPH# | Absolute lymphocyte count | 2.12±0.57 | 2.08±0.54 | 2.15±0.6 | 0.034 |

”

**（6）**It might be good to have your English proofread. Overall, it is understandable, but a review of spacing and similar details is necessary.

**Response:**

Thank you for your suggestion. We have conducted English proofreading of the manuscript to ensure the accuracy of expression.
