# Supplementary material for: Construction of an intelligent screening model for allergic rhinitis based on routine blood tests
Source: PLoS One. 2025 Dec 23;20(12):e0337561. doi: 10.1371/journal.pone.0337561 (PMC12725639; doi:10.1371/journal.pone.0337561)
Supplement: Attachment — Submitted filename: Reviewer Comments.docx [file pone.0337561.s002.docx]

Reviewer Comments:

I. Research Value and Innovation

Clear research significance with clinical application potential. The rising prevalence of Allergic Rhinitis (AR) and the limitations of current diagnostic methods (subjectivity, high cost, invasiveness) are well-established. This study builds an intelligent screening model using routine blood test data, enabling early screening without additional tests. This holds significant practical value for primary healthcare, aligning with trends in precision medicine and AI-assisted diagnosis.

Moderate methodological innovation. The ensemble voting approach integrating multiple feature selection techniques (filter, embedded, wrapper methods) with machine learning algorithms enhances feature selection reliability and model performance compared to single methods. The soft voting ensemble strategy, weighted by AUC to optimize classifier combination, is logically sound. Results demonstrate superior model performance over single algorithms (AUC=0.862, external validation accuracy 73.91%), providing methodological reference value.

II. Method Design and Implementation

Data Source and Preprocessing

Strengths: Data originates from a single center but has a substantial sample size (n=1295). Diagnostic criteria (combining symptoms and allergen testing) are clear. Grouping methods are reasonable.

Limitations:

Inclusion only of patients from Hohhot introduces geographical limitations potentially affecting model generalizability. This should be explicitly acknowledged in the Discussion.

Details of data preprocessing (e.g., missing value handling, outlier correction, feature standardization) are omitted, potentially impacting model stability. Supplementation is recommended.

Feature Selection and Model Construction

Strengths: The integrated hard voting method effectively cross-validates features across multiple strategies (retaining features with frequency ≥2). The final 16 retained features (e.g., eosinophil-related indices, RDW) align with AR immunopathological mechanisms (e.g., elevated eosinophils), demonstrating biological plausibility.

Limitations:

Specific parameter settings for the three feature selection methods (Mutual Information, LASSO, RF-RFE), such as the LASSO penalty coefficient or RF-RFE iteration count, are not detailed, hindering method reproducibility.

The rationale for selecting the top three base classifiers ("based on AUC value") lacks clarity regarding the threshold criterion. Supplementation with the AUC ranking results for all algorithms and statistical tests (e.g., Wilcoxon test) is recommended.

Model Evaluation

Strengths: Model generalizability is assessed using an external validation dataset. Multi-dimensional metrics (accuracy, AUC, etc.) are reported, and result visualization (confusion matrix) is clear.

Limitations:

The source, sample size, and feature distribution of the external validation set are not specified. Clarification is needed on whether it is an independent cohort or cross-center data.

Comparison with single algorithms is limited to AUC and accuracy. The absence of a calibration curve or Decision Curve Analysis (DCA) makes it difficult to comprehensively evaluate clinical net benefit.

III. Result Analysis and Discussion

Result Presentation

Strengths: The Venn diagram effectively illustrates the intersection of different feature selection methods. The model performance comparison figure (Fig 3) visually demonstrates the advantage of the ensemble method.

Limitations:

Feature importance ranking lacks discussion in the context of clinical significance (e.g., were eosinophil absolute count/percentage assigned the highest weights?). Supplementation with feature contribution analysis (e.g., SHAP values or Permutation Importance) is recommended.

Table 2 (Parameter Configuration) lacks complete presentation of some algorithm parameters (e.g., SVM kernel function, KNN neighbor count), hindering model replication.

The current figure legend for the confusion matrix (likely Fig 5) lists only "False positive," "False negative," and "Correct prediction" categories without specific numerical values or percentages. Adding this data would enhance clarity. Terminology must be consistent with definitions in the main text. Briefly define these terms in the legend or text. Color legends (■) must correspond precisely to chart colors. If figures are for potential B&W printing, use distinct patterns (e.g., hatching, dots) instead.

Regarding data presentation: The meaning of numbers like "136, 19, 29" below charts is unclear (possibly sample counts per category or other metrics). Explicit labeling is required to prevent misinterpretation. If sample counts, supplement with relevant statistical metrics (e.g., accuracy, recall) to bolster result credibility.

Formatting & Norms: Figure filenames (e.g., Fig5.tif) should follow journal style (e.g., "Figure5.tif" or "Fig5.png"). Ensure figure resolution meets journal requirements (typically ≥300 dpi).

Discussion Depth

Strengths: The Discussion objectively identifies study limitations (single data source, lack of symptom/exposure history integration) and proposes future directions (expanding data dimensions, incorporating multimodal information), presenting a clear logic.

Limitations:

Insufficient comparison with similar studies (e.g., Christo et al.'s 97.7% accuracy model). Analyze methodological differences (e.g., data type, algorithm choice) to clarify this model's specific applicability.

The impact of dynamic changes in blood routine indicators (e.g., seasonal fluctuations in seasonal AR patients) on the model is not explored and could be a valuable extension.

IV. Ethics and Writing Standards

Ethical Compliance. Mention of Institutional Review Board (IRB) approval is noted, but details regarding patient informed consent and data anonymization procedures are missing. Supplementation of the ethics statement details is required.

Writing and Formatting

Strengths: Well-structured manuscript. Abstract is concise. References cover recent relevant studies in ML and AR diagnosis.

Limitations:

Some figure/table labels are unclear (e.g., missing y-axis unit in Fig 3, inconsistent table numbering). Standardize figure/table formatting.

Redundant descriptions exist between the English abstract and main text (e.g., repeated steps of the ensemble voting in the Methods). Streamlining is advised.

V. Overall Recommendations

Major Revisions Required:

Data & Methods:

Supplement data preprocessing details (missing value handling, standardization methods) and feature selection parameter settings.

Clarify the source, sample size, and feature distribution of the external validation set, OR consider adding multi-center validation.

Supplement AUC rankings for all base classifiers and provide statistical test results to justify selecting the top three.

Authors must supplement charts/tables with specific data and explanations to ensure information is complete and easily understandable.

Check terminology and formatting for consistency with the journal's author guidelines.

Results & Discussion:

Add feature importance analysis (e.g., SHAP values) and interpret key features (e.g., EO#, RDW-SD) in the context of clinical literature and their biological significance.

Supplement calibration curve and decision curve analysis (DCA) to evaluate clinical utility.

Compare the advantages/disadvantages and innovation of this model against similar ML models for AR.

Provide deeper discussion of chart/table results, explicitly linking them to the research objectives.

Ethics & Formatting:

Enhance the ethics statement to include informed consent procedures and data privacy protection measures.

Correct figure/table labeling errors, unify table numbering, and eliminate redundant text.

Minor Revisions Suggested (Optional Optimizations):

Explore the interaction effect of demographic features (age, sex) with blood routine indicators on AR.

Compare other ensemble strategies (e.g., Stacking) with the soft voting method used here to further validate model robustness.

Summary: This study constructs an intelligent AR screening model based on routine blood test data. The methodology is sound, and the results hold clinical relevance. Enhancing data diversity, feature interpretability, and model evaluation dimensions would significantly strengthen its scientific rigor and persuasiveness. The authors are recommended to undertake a major revision addressing the points above before resubmission.
